# Fungicide Seed Coating Increases Emergence of Bluebunch Wheatgrass (*Pseudoroegneria spicata*) Under High-Fungal-Biomass Conditions

**DOI:** 10.3390/plants14050679

**Published:** 2025-02-22

**Authors:** Amber J. Johnson, Brad Geary, April Hulet, Matthew D. Madsen

**Affiliations:** Department of Plant & Wildlife Sciences, Brigham Young University, Provo, UT 84606, USA; brad_geary@byu.edu (B.G.); april_hulet@byu.edu (A.H.); matthew.madsen@byu.edu (M.D.M.)

**Keywords:** bluebunch wheatgrass, rangeland restoration, seed pathogens, soil pathogens, seed enhancement technology, seed coating, fungicide

## Abstract

Pathogenesis from soil- and seed-borne fungi can limit the survival and growth of native seeds and seedlings. Fungicides can combat fungal pathogens, but in some studies, fungicide treatments were ineffective at improving seedling emergence over untreated seed. Such studies suggest that low fungal presence due to dry conditions may be the cause of fungicide ineffectiveness in some years and sites. This study tested whether a fungicide treatment’s effectiveness is indeed related to the amount of fungi in the soil. We compared the emergence and biomass produced from *Pseudoroegneria spicata* seed that was uncoated, coated with no active ingredient, and fungicide-coated, across five soil treatments promoting different levels of fungal biomass. For uncoated seed, both percent emergence and total biomass of seedlings were highest in autoclaved soil and declined when fungi were present, but the level of fungus did not impact emergence or biomass for fungicide-coated seed. When grown in autoclaved, untreated, or low-fungus soils, percent emergence and total biomass from fungicide-coated seeds were not significantly different from uncoated seeds. However, in medium- and high-fungus soils, the percent emergence and total biomass from fungicide-coated seeds were more than two times greater than uncoated seed (*p* < 0.05). These results indicate that fungicide treatments can be effective at increasing restoration success for *P. spicata*, but the effectiveness of the fungicide treatment depends on the microbial environment of the planting site.

## 1. Introduction

Restoring native species after disturbance is becoming increasingly important for countering widespread ecosystem degradation across the globe [1,2,3]. Establishment of native species, however, can be negatively affected by various biotic and abiotic pressures [4,5]. In rangeland restoration efforts, typically fewer than 10% of seeds planted reach maturity [6,7]. Factors such as drought, pathogens, competition, soil crusting, and extreme temperatures may contribute to this low success [4]. These biotic and abiotic pressures limiting the establishment of native seeds need to be addressed to improve the success of restoration efforts [2,6]. A significant bottleneck impairing seeding success occurs between germination and emergence [8,9,10]. One factor contributing to this bottleneck is pathogenesis [11,12]. Research indicates that soil- and seed-borne pathogens have the potential to limit the survival and growth of native grass seeds and seedlings [9,13,14]. As climatic conditions change, pathogens may be an increasing concern for restoration due to seeds being more stressed by variable environmental conditions [5,9].

In the Great Basin region of the western United States (which encompasses parts of Nevada, Utah, Idaho, Oregon, and Wyoming), native seeds are often planted in autumn for various logistical reasons, but planting during this time allows seeds to be exposed to pathogens through the winter [1,15,16]. Autumn planting ensures that seeds are positioned to leverage the soil moisture accumulated from snow or rain during the winter, allowing for seed germination and plant growth in the spring [16]. This is important in the Great Basin since these areas are water-limited and most of the precipitation comes as winter snow [16]. However, cool, moist soil conditions are highly conducive to pathogens, and seeds that overwinter in these environments are susceptible to pathogenesis [9,10,15]. In the Great Basin, autumn-sown seeds are exposed to these conditions for several months before they can emerge from the soil in the spring [8,9,10]. A potential mechanism to increase native seed emergence and survival over the winter is to protect seeds from pathogens. Seed coatings may help overcome this limitation by providing a protective shell with anti-pathogen ingredients close to the seed and systemic protection within the seed [17].

Chemical fungicides are one method of combatting fungal pathogens [11,18]. Because a variety of seed-borne and soil-borne pathogens can negatively impact native seeds, a mix of different fungicides can be used to target a variety of known pathogens [10,19]. Research on *Pseudoroegneria spicata* (Pursh) Á. Löve has identified several pathogens frequently associated with this species [9]. Subsequently, research targeting these pathogens showed that a mix of fungicides incorporated into a seed coating improved seed germination, seedling emergence, and plant growth [10,20]. Increases in seedling emergence can be over 300% with fungicide seed coating, but success varies by site and year [10,20].

Although fungicide treatments can be effective, some trials have demonstrated that a fungicide treatment is not always effective at improving seedling emergence over untreated seed [10,20,21,22]. A theory postulated to explain why fungicide treatments are ineffective in some cases suggests that during specific years and locations, dry conditions led to fewer fungal pathogens in the soil [10,20,22]. Moisture is a key influence on fungal occurrence [23,24,25], and fungal populations, including pathogenic fungi, generally decrease as moisture decreases and increase with increasing moisture [26,27]. Rogers tracked mycelial strands across temperature and moisture gradients and observed that the strands degraded rapidly under high temperatures or drying [26]. If conditions are dry, the patterns explained by Rogers, Griffin, and Shields et al. suggest that there may be fewer fungi in the soil [23,26,27]. Under reduced pathogen pressure, the need, and thus the efficacy, of fungicide treatments may be diminished, whereas during wetter years with increased soil fungi, fungicide treatments are expected to be more potent.

The objective of this study was to test if the effectiveness of fungicide seed coating is related to the amount of fungi in the soil when moisture is not limited. We investigated the impact of fungal load on the performance of uncoated and fungicide-coated *P. spicata* seeds. We compared seedling emergence and plant biomass from uncoated and fungicide-coated seeds sown in various fungal conditions, ranging from autoclave-sterilized soil to untreated soil, and soils with increasing levels of fungal inoculation. We predicted that plant emergence and biomass from uncoated seed would decrease more than fungicide-coated seed with increasing fungal pressure. We further hypothesized that the emergence and plant biomass from fungicide-coated seed would only be higher than uncoated seed in soils with higher fungal loads.

## 2. Results

### 2.1. Fungal Biomass

**Figure 1:**
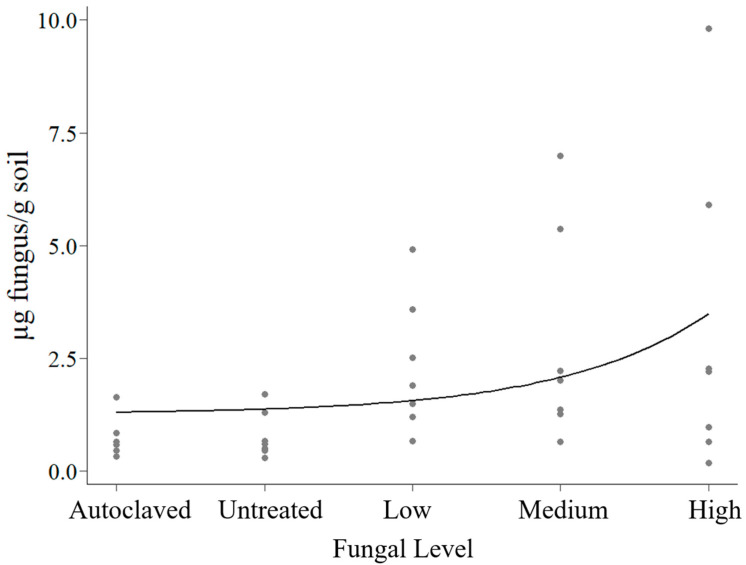
Fungal biomass in micrograms of fungus per gram of soil by fungal inoculation level. The trendline shows the linear regression after the fungal biomass was log transformed.

There was a positive trend between fungal inoculation level and fungal biomass (*p* = 0.005; Figure 1). Fungal biomass increased from the autoclaved soil to the untreated soil and through the increasing levels of fungal inoculated soils.

### 2.2. Seedling Emergence

**Figure 2:**
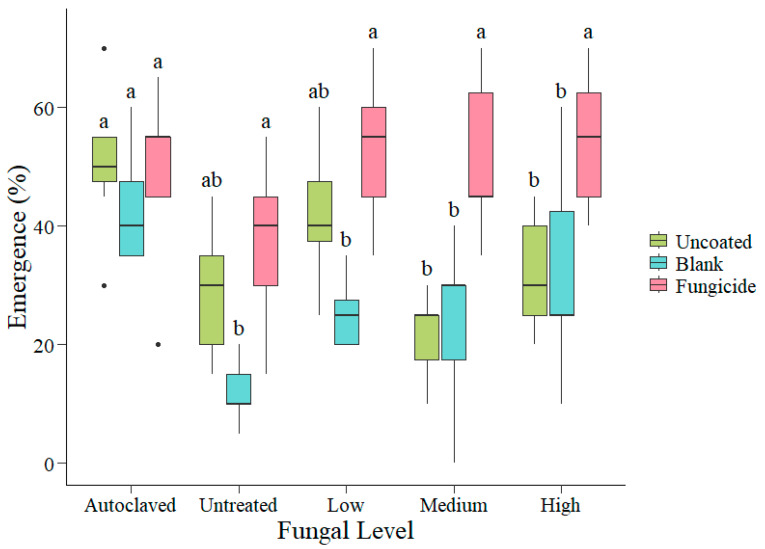
Percent emergence of uncoated, blank, and fungicide-coated seed grown in a range of fungal inoculum levels. Letters indicate significant difference at the *p* < 0.05 level within fungal inoculum levels using the Tukey method. Whiskers on each box represent the highest 25% and the lowest 25% of the data. Black dots represent outliers.

Percent emergence from uncoated seeds was greatest in autoclaved soil ($\bar{x}$ = 50.7%) with 2 times higher emergence than both untreated soil ($\bar{x}$ = 28.6%; *p* = 0.04) and medium-fungus soil ($\bar{x}$ = 21.4%; *p* < 0.001; Figure 2). Emergence was not significantly different for uncoated seed between autoclaved soil and low-fungus soil (*p* = 0.99) or high-fungus soil (*p* = 0.17). Emergence was also not significantly different between uncoated and blank-coated seeds at any levels of inoculum (*p* > 0.27; Figure 2). Emergence for fungicide-coated seeds did not differ significantly across fungal levels (*p* > 0.27). In autoclaved, untreated, and low-fungus soils, emergence from fungicide treatments was not different from emergence of uncoated seedlings (*p* > 0.91; Figure 2). However, emergence from fungicide-coated seeds was 8 times greater than uncoated seed in medium-fungus soils (uncoated $\bar{x}$ = 21.4%; fungicide-coated $\bar{x}$ = 52.1%; *p* < 0.001) and 2 times greater in high-fungus soils (uncoated $\bar{x}$ = 32.1%; fungicide-coated $\bar{x}$ = 54.3%; *p* = 0.04; Figure 2).

### 2.3. Total Above-Ground Biomass

**Figure 3:**
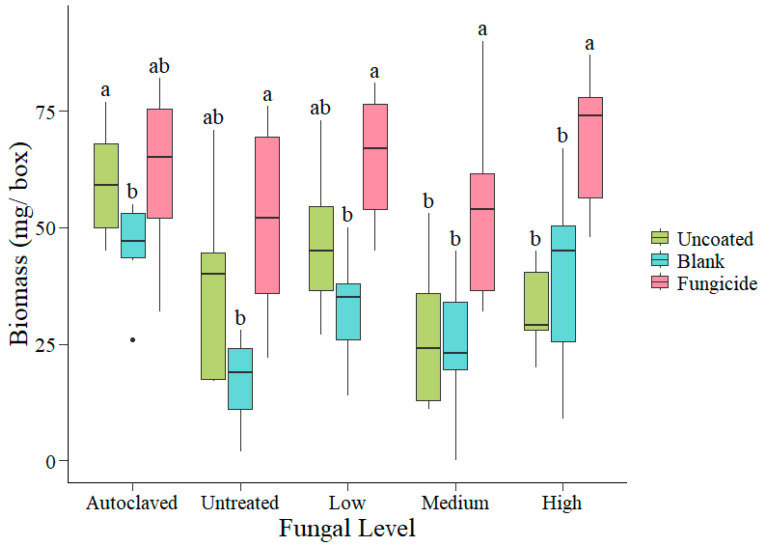
Total biomass per box for uncoated, blank, and fungicide-coated seed grown in a range of fungal inoculum levels. Letters indicate significant difference at the *p* < 0.05 level within fungal inoculum levels using the Tukey method. Whiskers on each box represent the highest 25% and the lowest 25% of the data. Black dot represents an outlier.

Total biomass per box showed similar responses to percent emergence. Uncoated seedling biomass was greatest in autoclaved soil ($\bar{x}$ = 0.06 g), with 2 times greater biomass than both medium- ($\bar{x}$ = 0.027 g; *p* = 0.003) and high-fungus soils ($\bar{x}$ = 0.033 g; *p* = 0.05; Figure 3). Uncoated seedlings had 1.3 times the biomass of blank-coated seedlings in autoclaved soil (uncoated $\bar{x}$ = 0.06 g; blank-coated $\bar{x}$ = 0.046 g; *p* = 0.04) but were not significantly different from blank-coated seedlings grown in any other level of inoculum (*p* > 0.15; Figure 3). For fungicide-coated seedlings, biomass did not differ across levels of inoculum (*p* > 0.66; Figure 3). In autoclaved, untreated, and low-fungus soils, biomass from fungicide treatments was not significantly different from uncoated seedlings (*p* = 1; 0.76; 0.53, respectively). However, biomass of fungicide-coated seedlings was 2 times greater than uncoated seed in both medium- (uncoated $\bar{x}$ = 0.027 g; fungicide-coated $\bar{x}$ = 0.053 g; *p* = 0.001) and high-fungus soils (uncoated $\bar{x}$ = 0.033 g; fungicide-coated $\bar{x}$ = 0.068 g; *p* = 0.05; Figure 3). Fungicide-coated seedlings had significantly more biomass than blank seedlings in untreated (blank $\bar{x}$ = 0.017 g; fungicide-coated $\bar{x}$ = 0.052 g; *p* = 0.002), low- (blank $\bar{x}$ = 0.032 g; fungicide-coated $\bar{x}$ = 0.065 g; *p* = 0.004), medium- (blank $\bar{x}$ = 0.025 g; fungicide-coated $\bar{x}$ = 0.053 g; *p* = 0.027), and high-fungus soils (blank $\bar{x}$ = 0.039 g; fungicide-coated $\bar{x}$ = 0.068 g; *p* = 0.017; Figure 3).

## 3. Discussion

Uncoated seed of *P. spicata* had its best seedling emergence and total biomass in autoclaved soil but declined in soil with fungi. Fungicide-coated seed was not significantly different than uncoated seed in autoclaved soil but was not impacted by increasing soil fungal levels like the uncoated seed and blank seed. This led to the fungicide-coated seed performing significantly better than uncoated seed at the medium and high soil fungal levels. These results may help to explain why previous research has reported variable success rates for fungicide seed treatments [10,21,22,28]. Fungal populations generally decline under dry conditions [23,26,27], and studies have proposed that low pathogen presence may be the reason fungicide-treated seed did not differ from untreated seed in establishment and growth [10,21,28]. Based on the findings of this study, we expect fungicide seed coating to improve seedling emergence and biomass in years and locations with ample precipitation but not in dry sites where the fungal population has declined due to desiccation [26,29].

Although blank-coated seed performance was similar to uncoated seed for most treatments, blank-coated seed had lower total biomass than uncoated seed in autoclaved soil. Emergence, however, was not significantly different between blank-coated and uncoated seeds in autoclaved soil. As this coating had no active ingredient, this indicates that when there are low fungal loads in the soil, seed coating alone may have a slightly negative effect on the growth of *P. spicata*. In most cases, however, seeds coated with a blank coating performed the same as the uncoated seeds. When more fungal pathogens were present, coating without an active fungicide ingredient did not benefit seedling emergence and growth, indicating the fungicide has an influence on the fungal pathogen seed interaction. For situations where higher levels of fungal pathogens are present, blank coating is not an effective treatment, but coating with a fungicide is effective.

It is important to note that our low-, medium-, and high-level inoculated soil had the added organic matter of the seeds used to inoculate the boxes with fungi, which may have affected the observed results. The main purpose of organic matter in our experiment was to act as an inoculum, because the seeds likely carry pathogens, and as an energy source for the microbes [25,27]. Microscope analysis indicated an increase in fungi from our autoclaved and untreated soils through our inoculated soils, fulfilling the purpose of the seeds as a fungal inoculum and energy source. Similar research has shown that a fungicide treatment has a greater effect when levels of organic matter are higher [20]. The organic matter may have also influenced bacteria, nutrients, and moisture availability, but this study did not measure these factors [15,21,30].

Our study indicates that seed coatings containing fungicides can be effective at increasing restoration success for *P. spicata*, but the effectiveness of the fungicide depends on the microbial environment of the planting site [10,20,22]. As the abiotic and biotic conditions at a restoration site greatly influence treatment and restoration success, land managers should consider both abiotic and biotic conditions when planning a restoration project [4,31,32]. Laboratory trials like ours can test how restoration technologies work under a variety of conditions [33]. Understanding how restoration techniques perform under various conditions is crucial for guiding restoration practitioners in applying restoration techniques.

## 4. Materials and Methods

### 4.1. Model Species

Given previous evidence of increased emergence with fungicide seed coatings for *P. spicata* var. Anatone, we selected this cultivar to test the effectiveness of fungicide seed coatings across varying fungal populations [10]. *P. spicata* is an important species due to its forage value for wildlife and livestock and its ability to establish and grow in drought conditions [34]. This species is especially useful for restoration as its seed can be successfully cultivated in plant production plots, harvested, and sown using rangeland seeding equipment [34]. Seed for this research was obtained from the Utah Division of Wildlife Resource’s Great Basin Research Center (Ephraim, UT, USA). The seed had 93% purity and a 93% germination rate.

### 4.2. Seed Treatments

We evaluated two different seed coatings against uncoated seeds. Seeds were either treated with a fungicide coating, coated without active ingredients (identified as “blank”), or left uncoated. In the fungicide coating, we applied a mixture of four different fungicides to target known pathogens of native seeds, such as *Fusarium* spp., *Sclerotinia homoeocarpa*, *Gibberella fujikuroi*, and *Verticillium dahlia* [9,29]. These fungicides were Apron, Dividend, Dynasty, and Maxim with active ingredients Mefenoxam, Difenoconazole and Mefenoxam, Azoxystrobin, and Fludioxonil, respectively (Syngenta, Basel, Switzerland). Mefenoxam targets oomycetes (e.g., *Phytophthora*, *Pythium*, etc.), while Difenoconazole, Azoxystrobin, and Fludioxonil are all broad-spectrum fungicides that target a variety of ascomycetes, basidiomycetes, deuteromycetes, and oomycetes [35]. All fungicides were applied at 167% label rate for cereal grasses, but well below the maximum allowable amounts per unit area [10]. This rate was chosen for consistency following a previous study which used the same seed coatings in a field trial [10].

Fungicide coating was applied to 200 g batches of seed in a two-step process using a 31 cm rotary seed coater (Universal Coating Systems, Independence, OR, USA) following standard seed coating protocols [17,36]. Fungicide was applied during the first step in a solution of Agrimer SCP II binder (Ashland Inc., Covington, KY, USA; Table 1). The fungicide solution was applied directly to the seed with the liquid injected with a syringe onto the seed coater’s atomizer disk. In the second step, we gradually added calcium carbonate powder (Clayton Calcium, Parma, ID, USA) directly over the seed while pumping Agrimer SCP II binder onto the seed via the atomizer disk (Table 1). This second application formed a protective, hardened coating around the seed, reducing fungicide leaching and protecting it against environmental and climatic conditions.

We followed the same procedure as that for the fungicide coating to coat the blank treatment but without adding fungicides (Table 1). The blank treatment served as a procedural control to observe the effects of the coating without active ingredients. All three seed treatments were dried at room temperature (~21 °C) on a forced-air dryer for approximately 10 min (Universal Coating Systems, Independence, OR, USA).

### 4.3. Soil Inoculation

We conducted an in vitro laboratory trial under five soil fungal levels to examine the effectiveness of seed coatings under varying pathogen populations. Soil was collected from a degraded rangeland site near Santaquin, UT, USA (39.9073, −111.8163) and was classified as a stony loam [37]. Rocks and debris were removed by passing the soil through a 4.75 mm sieve. Soil was placed in 7 cm × 7 cm × 10 cm polycarbonate Magenta plant tissue culture boxes (Plantmedia, Dublin, OR, USA) with four 4 mm holes drilled in the bottom for drainage. We placed a second Magenta box over the box containing soil to maintain moisture and prevent the transfer of fungal spores. We treated the soil to both decrease and increase fungal populations to create five soil fungal levels. The lowest of the five fungal levels was created by autoclaving soil for 12 h to kill most fungi (hereafter “autoclaved”). The second fungal level consisted of soil where the natural fungal community was not manipulated (hereafter “untreated”). The final three fungal levels (low, medium, and high) were created by increasing fungal levels using five rounds of inoculation as described below.

We seeded 150 *P. spicata* seeds within the top 5 mm of soil in each Magenta box, allowing us to use the fungal inoculum naturally present on the seeds to inoculate the soil. Each box was watered by saturating the soil from the bottom up by placing them in trays filled with deionized water. All trays containing the Magenta boxes were placed in a growth chamber (Environmental Growth Chambers, Chagrin Fall, OH, USA) programmed to alternate between 11 °C and 6 °C on a 12 h light/dark cycle. After one week, germinated seeds were cut in half with scissors to kill the plants, and another 150 seeds were added to the soil surface. This process was repeated until we had seeded the boxes four times (600 seeds total). After the fourth addition of seeds, we continued to cut germinated seeds weekly for three weeks to kill all plants. Following the seven-week inoculum build-up, a fifth round of *P. spicata* seeds was planted in the boxes, but at three rates to achieve a low, medium, and high fungal level, 75, 150, and 231 seeds, respectively. These seeding rates were determined with preliminary studies that monitored the growth of *P. spicata* in inoculated boxes under different seeding rates. Boxes were again watered weekly as described above, and germinated seeds were cut in half each week for three weeks.

### 4.4. Study Design

After increasing the fungal pathogen inoculum in the Magenta boxes, each box was planted with 20 *P. spicata* seeds. The 20 seeds planted were either uncoated, coated with a blank coating, or coated with fungicide, according to the treatment for each box. Toothpicks were used to mark where each seed was planted. The study was designed as a randomized complete block split-plot design with seven blocks, each consisting of 15 boxes. The blocks were randomly divided by fungal level to prevent the transfer of spores between fungal inoculum levels. The 3 seed treatments (uncoated, blank, and fungicide) were randomized within each fungal level (5 fungal levels × 3 seed treatments × 7 blocks = 105 boxes). All boxes were then placed in a 4 °C controlled environmental room (R.W. Smith and Company, San Diego, CA, USA) for three weeks to mimic winter field conditions and provide time for pathogens to attack the seeds. Boxes were then moved back to the growth chamber and watered weekly as described previously. After three weeks in the growth chamber, we counted the number of toothpick-marked seedlings per box and harvested the above-ground biomass. Above-ground biomass was oven-dried for four days at 60 °C and then weighed. Many fungal pathogens cause stunted growth and etiolation in addition to seed death. The percent of seedlings that emerged allowed us to examine seed death, while above-ground biomass allowed us to measure additional growth effects.

### 4.5. Fungal Biomass Quantification

To assure that fungal biomass varied across different fungal levels, we used an i4 infinity microscope with a 40× objective lens (LW Scientific, Lawrenceville, GA, USA) to quantify fungal biomass per gram of soil [38,39,40]. Three grams of soil from the top 10 mm of soil were collected from each box of a given fungal level in each block (three boxes per fungal level per block). These three samples were thoroughly mixed, and a 1 mL subsample of the soil mixture was then diluted with 9 mL of water. A drop of the diluted solution was placed on a microscope slide, covered with a coverslip, and examined to measure the length and width of all fungal hyphae [39]. Hyphal lengths and widths were used to calculate fungal volume [39,41], which was then converted to biomass using the conversion factor recommended by Van Veen and Paul:

$$m=\left(\frac{\pi {\left(\frac{w}{2}\right)}^{2}l}{1,000,000}\right)0.33$$

where *m* denotes biomass in μg, *w* represents the width of the hyphae in μm, and *l* represents the length of the hyphae in μm [42].

### 4.6. Statistical Analysis

The relationship between relative fungal biomass and fungal level was analyzed using linear regression in R version 4.4.1 [43]. Because the fungal biomass data were skewed, as no fungal biomass could be less than zero, we log transformed these data before performing the linear regression [44]. The total above-ground biomass of the boxes and the percentage of emerged seedlings were also analyzed using general linear mixed-effect models. For these models, seed treatment, soil fungal level, and the interaction between seed treatment and soil fungal level were defined as fixed effects. For all models, block was defined as a random effect to account for variation across blocks. We constructed models using the ‘lmer’ function of the ‘lme4’ package version 1.1-36 in R [43,45]. Residuals were checked for normality and equal variance. Pairwise comparisons were then conducted using the Tukey method with the ‘emmeans’ package in R [46].

## Figures and Tables

**Table 1:** Fungicide and blank seed coating recipes.

Treatment	Agrimer SCP II	Calcium Carbonate	Apron (Mefenoxam)	Dividend (Difenoconazole and Mefenoxam)	Dynasty (Azoxystrobin)	Maxim (Fludioxonil)
	---------------------------------------------------- grams ----------------------------------------------------------					
Fungicide	130	350	0.163	1.088	0.09	0.044
Blank	130	350	0	0	0	0

## Data Availability

Data are available on the Brigham Young University Scholars Archive https://scholarsarchive.byu.edu/data/60/ (accessed on 29 December 2023).
